# Time-dependent application strategies of selenium Nanofertilizer to enhance biofortification in rice and wheat

**DOI:** 10.1016/j.fochx.2025.103174

**Published:** 2025-10-16

**Authors:** Xin Wang, Bilal Hussain, Jiapan Lian, Xiaoping Xin, Tong Zou, Xiwei Huang, Liping Cheng, Hongyu Yu, Zhenli He, Xiaoe Yang

**Affiliations:** a Ministry of Education (MOE) Key Laboratory of Environmental Remediation and Ecosystem Health, College of Environmental and Resources Sciences, Zhejiang University, Hangzhou 310058, China; b State Key Lab for Conservation and Utilization of Subtropical Agro-Bioresources, Guangxi Key Lab of Sugarcane Biology, Guangxi University, Nanning 530004, China; c School of Natural Resources, Division of Plant Sciences and Technology, University of Missouri, MO 65211, USA; d Department of Soil, Water and Ecosystem Sciences, Indian River Research and Education Center, University of Florida-IFAS, Fort Pierce, FL 34945, USA

**Keywords:** Selenium nanoparticles, selenium speciation, HPLC-ICP-MS, SP-ICP-MS, Food safty

## Abstract

Understanding the effects of different fertilization timing on crop selenium biofortification is of great importance. This field study evaluated the effects of Se nanoparticle application at different growth stages on crop yield, Se accumulation, and Se speciation. Se NPs application at the jointing stage significantly increased grain yield (up to 10.2 % in rice, 8.4 % in wheat) and Se concentration (9.7-fold in rice, 21-fold in wheat). Rice showed higher Se uptake efficiency, while wheat exhibited greater translocation of Se from leaves to grains. Notably, Se NPs were absent from grains in both rice and wheat, ensuring food safety. Selenomethionine was the dominant Se species in both grains (>62 %), unaffected by fertilization timing. Delayed fertilization increased Se retention in the wheat inner fraction, enhancing Se recovery during flour processing. These findings offer practical guidance for optimizing Se biofortification strategies to improve grain Se content and nutritional quality.

## Highlights

• Application of Se NPs at booting stage maximizes crop yield and Se concentration.
• Rice excels in fertilizer use efficiency but not in grain nutrient use efficiency.
• Delayed fertilization increases Se recovery from refined wheat flour.
• Se accumulates in cereals not in particulate form but in ionic form - mostly SeMet.
• Se Bioaccessibility of cereals is positively correlated with total Se concentration.

## Graphical abstract

**Figure:**
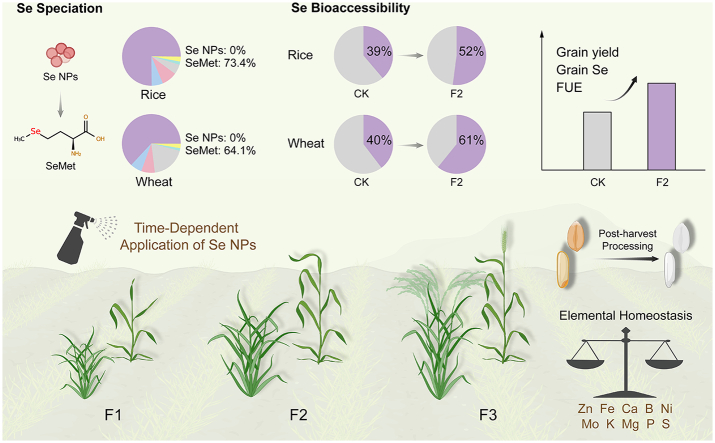

## 1 Introduction

Crop selenium (Se) biofortification represents an effective strategy to enhance dietary Se intake and mitigate Se deficiency in the global population. Rice and wheat are two of the most vital food crops globally, playing significant roles in both human diets and agricultural production(Farooq et al., 2022; Gupta & Gupta, 2017). Rice cultivation spans across various regions worldwide, including Asia, Africa, the Americas, and Australia(Londo, Chiang, Hung, Chiang, & Schaal, 2006; Wang et al., 2025; Yu et al., 2020). Meanwhile, wheat serves as an indispensable staple food for 40 % of the world's population(Dinh et al., 2019; Gashu et al., 2021; Gupta & Gupta, 2017). The extensive cultivation and consumption of rice and wheat make them the prime choice for Se biofortification.

Beyond total Se levels in the grains, the nutritional value of Se is determined by its distribution and speciation, (X. Wang et al., 2024; Finley, 2006; Lyons, Genc, Stangoulis, Palmer, & Graham, 2005). Both rice and wheat grains are commonly subjected to polishing during processing, which removes the mineral-rich bran and leads to a loss of nutrients(Lian et al., 2024; Wang et al., 2024). Se in the grain primarily derives from the redistribution of pre-existing Se from leaves, whereas the developmental timing of grain components (including bran and endosperm) is not uniform(Liang, Zhou, & Chen, 2025). Owing to this spatiotemporal asynchrony, the deposition and fixation of Se within different grain tissues may be sensitive to the timing of its arrival. Therefore, varying the timing of Se application may alter the spatial distribution of Se within the grain. In addition, Se speciation plays a vital role in its bioavailability in humans(X. Zhou et al., 2024; Ulhassan et al., 2023; Qin et al., 2023). However, there remains disagreement regarding the primary speciation of Se in crops. Some studies have identified selenomethionine (SeMet) as the predominant Se species in both rice and wheat grains(Di et al., 2023; Zeng et al., 2023), other research has suggested that selenocysteine (SeCys) maybe the major form in rice(Dai et al., 2019) and wheat(Yeasmin et al., 2025). Therefore, study on Se Speciation in crops remains to be explored. In particular, whether fertilization timing modulates Se distribution and speciation is largely unknown.

Among current Se biofortification strategies, foliar application of Se NPs has proven to be more effective compared to inorganic Se or soil application(Chen et al., 2024; Wang et al., 2022; Zhou et al., 2021). However, a limitation is the need for multiple applications at different growth stages.(Dai et al., 2019; Deng et al., 2017; Yuan et al., 2022). This frequent reapplication increases labor, time, and resource costs associated with fertilization. More importantly, the nanoscale dimensions of Se NPs enable rapid penetration into plants, their subsequent transport in grains raise concerns(Bolan et al., 2024; Hawkins et al., 2018). Once inside the leaf, Se NPs can translocate and simultaneously undergo time-dependent dissolution(Wang et al., 2023; Wang et al., 2025). Accordingly, the timing of application is likely to modulate Se NPs accumulation and therefore warrants investigation. Furthermore, Previous Se biofortification studies have focused on a single crop, lacking side-by-side comparisons of different crops under comparable application conditions. Therefore, developing an optimal, tailored fertilizer application strategy for specific cereals is crucial for reducing input costs and enhancing agricultural productivity.

Hence, we conducted a field experiment within a rice-wheat rotation system with foliar application of Se NPs at different growth stages of crops. The objectives were: 1) to examine the effects of different fertilization timing on crop growth, nutritional quality, and Se accumulation; 2) to investigate the distribution, speciation, and bioaccessibility of Se in cereals, and 3) to analyze the accumulation of Se NPs in plant tissues using single-particle inductively coupled plasma mass spectrometry (SP-ICP-MS). This research provides a scientific basis for developing effective Se biofortification strategies in rice and wheat.

## 2 Materials and methods

### 2.1 Synthesis and characterization of Se NPs

Se NPs were synthesized using the chemical reduction method(Lin & Chris Wang, 2005) with some modifications. Briefly, Sodium thio-sulfate pentahydrate was used as the reducing agent, and selenium dioxide as the oxidizing agent, with 0.01 M sodium dodecylsulfate serving as the surfactant. The reducing and oxidizing agents were mixed at final molar concentration ratios of 2:1 and stirred for 30 min (min). After standing for 48 h, excess reagents were removed by centrifugation 3 times in deionized (DI) water. The resulting Se NPs were characterized using several techniques, including scanning electron microscopy (SEM) (G300, Zeiss, Germany) combined with energy-dispersive spectroscopy (EDS), transmission electron microscopy (TEM) (JEM -1400, JEOL, Japan), selected area electron diffraction (SAED) (JEM-2100plus, JEOL, Japan) and dynamic light scattering (DLS) (Zetasizer Pro, Malvern, UK).

### 2.2 Field trial and experimental design

The field experiment was conducted in a rice-wheat rotation area in Zhejiang Province, China. To avoid the preceding crop effect, rice and wheat were planted in different areas of the same field. The soil was acidic paddy soil (pH 5.4) with a Se concentration of 0.16 mg/kg. More basic characteristics of the experimental soil are presented in Table S1. During the cultivation period, air temperature ranged from −9 to 40 °C (mean = 22 °C); daily precipitation peaked at 122.45 mm (mean = 4.75 mm/day), and relative humidity varied between 33 % and 97 % (Fig. S1). The trial was conducted in a randomized complete block design (RCBD) with each plot size of 4.5 m^2^ (1.5 m × 3 m). The growing seasons for rice and wheat were June–November and December–May, respectively. The foliar application was carried out at late jointing stage (F1), booting stage (F2) and filling stage (F3), respectively. The application dosage was referred to previous studies(Hussain et al., 2020; Yan et al., 2024) and was set at 30 mg/L × 300 mL, equivalent to 20 g/ha. The control group (CK) was treated with an equal volume of DI water. Each treatment had six replications. All cultural practices were carried out according to local customs. Upon maturity, plant tissues were collected, thoroughly washed to remove any surface residues, dried at 50 °C, and the final weight was recorded. For mineral element distribution analysis, a portion of the grains was processed with a pearling mill (JNNJ3B, Taizhou, China), separated into inner and outer fraction in a conventional production ratio of 3:7 (wheat) and 2:8 (rice).

### 2.3 Analysis of elements

A 0.2 g portion of ground samples was pre-digested in 5 mL HNO_3_ overnight and then further digested in a microwave digestion system (WX-8000, PreeKem), The microwave digestion program was set to 100 °C for 10 min, followed by 120 °C for 40 min. After digestion, the samples were heated on the graphite furnace until the liquid volume reached to approximately 1 mL. The digesta was then diluted with DI water and filtered through a 0.22 μm cellulose acetate membrane. The filtrate was quantified by inductively coupled plasma mass spectrometry (ICP-MS) (Plasma Quant MS, Analytik Jena, Germany) or inductively coupled plasma optical emission spectroscopy (ICP-OES) (ICP6000, Thermo Fisher Scientific, U.K.). Blanks, spiked samples, and standard reference materials (Se-enriched rice, GBW(E)100,700) were included to validate the digestion and analytical methods.

### 2.4 Measurements of phytate, protein, soluble sugars, and starch in grains

The phytic acid content in rice and wheat grains was determined following extraction, using the method outlined in our previous work(Lian et al., 2024). 1.0 g of grain flour was added to a 50 mL centrifuge tube containing 20 mL of a 10 % HCl-Na_2_SO_4_ solution (10 % *w*/*v* Na₂SO₄ prepared in 1.2 % w/v HCl). The mixture was first vortexed vigorously for 1 min and then extracted for 2 h (200 rpm, 25 °C) before centrifugation (4000 *g*, 20 min). The supernatant was collected, mixed with an equal volume of 15 % trichloroacetic acid (TCA) solution, and incubated at 4 °C for 2 h. The reaction solution was centrifuged (10,000 *g*, 10 min) and the pH was adjusted to 6–6.5 with 0.75 M sodium hydroxide. Supernatant (0.1 mL) were collected, mixed with 1.4 mL deionized water and then 0.5 mL ferric chloride-sulfosalicylic acid solution were added on it. Finally, the mixture was reacted for 20 min and its absorbance was measured at 500 nm using a microplate reader (Epoch2, BioTek, Thermo Fisher Scientific, USA). The concentration of phytic acid was calculated using the standard curve of sodium phytate.

And the concentrations of soluble sugars and starch content in grains were measured using the anthrone colorimetric method. 100 mg of ground grain was homogenized in 8 mL of 80 % ethanol and then extracted in a water bath at 80 °C for 30 min. The supernatant was collected by centrifugation at 4000 rpm for 5 min at room temperature. The extraction was performed twice following the same steps and the three supernatants were pooled for analysis of soluble sugars. For starch, the residue was then heated with 2 mL of deionized water in a 95 °C water bath for 15 min, and then 2 mL of H2SO4 was added into each extract for another 15 min. The extracts were centrifuged at 4000 rpm for 10 min to obtain the supernatant and the residue was extracted again with 50 % H2SO4. After colored with anthrone solution, the supernatant was measured at 620 nm by microplate reader (Epoch2, BioTek, USA). A standard curve is plotted using a glucose standard solution.

### 2.5 Analysis of NPs residues on leaf surfaces

At maturity, crop leaves were collected for qualitative and quantitative analysis of Se residues on leaf surfaces, to assess the uptake of NPs by the crops. The samples were dried at 50 °C and plated with Pt using a Hitachi ion sputter coater before SEM analysis, and no additional treatments were applied to avoid movement of NPs. In addition, fresh leaves of uniform size were collected and subjected to ultrasonic treatment in 100 mL of DI water to elute any potentially remaining Se NPs. The concentration of Se ion and Se NPs was then quantified using single particle inductively coupled plasma mass spectrometry (SP-ICP-MS).

### 2.6 SP-ICP-MS measurement of Se NPs in plant tissues

Nanoparticles in grain and leaves of crops were extracted by enzymolysis and detected by SP-ICP-MS (EXPEC 7350 in a single particle mode). Following the method described by Dan et al. (2015), 0.025 g of ground grain samples were homogenized with 8 mL DI water, then 2 mL of the enzyme solution (0.05 g of Macerozyme R-10 dissolved in 2 mL of DI water) was added. The pH was adjusted to 5.5 using HCl and NaOH, which is within the optimal pH range (3.5–7.0) for the enzyme, as specified by the manufacturer. The samples were then shaken for 24 h at 37 °C and 150 rpm/min. After digestion, the samples were allowed to settle for 1 h and the supernatant was diluted using DI water for SP-ICP-MS analysis. An 80 nm Au NPs standard was used for quality control. The operating parameters and information for the instrument are detailed in Table S2.

### 2.7 Speciation analysis of grain Se

The speciation of Se in grains, including *Se*-methyl-selenocysteine (MeSeCys), selenocysteine (SeCys_2_), selenomethionine (SeMet), selenite (Se(IV)), and selenate (Se(VI)) was determined by High Performance Liquid Chromatography-Inductively Coupled Plasma Mass Spectrometry (HPLC-ICP-MS) following enzymatic digestion. Briefly, 0.2 g of ground grain was added to 10 mL of DI water and subjected to ultrasonication for 30 min. Then, 25 mg of protease E was added, and the mixture was then incubated 37 °C with shaking for 24 h, filtered through a 0.22 μm membrane filter, and appropriately diluted before analysis. Se standards for MeSeCys, SeCys_2_, SeMet, Se(IV), and Se(VI) were used to validate the enzymatic and analytical methods.

### 2.8 Bioaccessibility of Se in grains

Se bioaccessibility (BAC) in rice and wheat grains was determined by the physiologically based extraction test (PBET) in vitro assay(Lian et al., 2024) with some modifications. Ground grain (0.5 g) was placed into a 50 mL polypropylene tube with 2 mL of DI water and subjected to a 100 °C water bath for 3 min to simulate cooking. After cooling, the sample was mixed with 30 mL of gastric solution (1.25 g pepsin, 0.5 g malate, 0.5 g citrate, 420 μL lactic acid and 500 μL acetic acid made up to 1 L with DI water, then adjusted the pH to 2.0 with 12 M HCl). The mixture was then shaken for 1 h in a thermostatic bath at 37 °C with 150 rpm. The solution was then neutralized to pH 7 with saturated NaHCO_3_, and then bile salts (52.5 mg) and pancreatin (15 mg) were added into each tube to stimulate the intestinal phase. The mixture was then shaken for additional 4 h in a thermostatic bath at 37 °C with 150 rpm. A 5 mL aliquot was extracted from the intestinal incubation and centrifugated at 4000 rpm for 10 min. The supernatants were filtered through 0.22 μm nylon membrane and refrigerated at 4 °C before analysis. The bioaccessibility of Se was calculated as follow:

$$\mathrm{BAC}\left(\%\right)=\frac{\mathrm{Se}\;\mathrm{in digestive fluid}}{\mathrm{Total}\;\mathrm{Se}\;\mathrm{in grain}}$$

### 2.9 Data analysis

Harvest index (HI), translocation factor (TF), fertilizer use efficiency (FUE), grain nutrient use efficiency (GUE), recovery rate, and estimate of daily Se intake (EDI) were calculated. The detailed formulas and descriptions are provided bellow:

Harvest index (HI) was calculated as the ratio of harvested grain dry weight (M_g_) to total plant biomass (M_t_):

$$\mathrm{HI}=\frac{M_{g}}{M_{t}}$$

Translocation factor (TF) of Se between tissues was calculated as follows:

$${\mathrm{TF}}_{j-i}=\frac{c_{i}}{c_{j}}$$

Where i and j represent plant tissues, and c represents Se concentration.

Fertilizer use efficiency (FUE) was estimated based on the total Se content (Ct) in wheat tissues, minus the amount of Se absorbed from the soil as indicated by the Se content in the whole plant of CK (Ct_CK_), divided by the total dosage (TD) of Se fertilizer applied per hectare (20 g/ha). Ct_g_, Ct_s_, Ct_l_ and Ct_r_ indicate content of Se in grain, stem, leaf and root, respectively.

$$\mathrm{FUE}=\frac{{\mathrm{Ct}}_{g}+{\mathrm{Ct}}_{s}+{\mathrm{Ct}}_{l}+{\mathrm{Ct}}_{r}-{\mathrm{Ct}}_{\mathrm{CK}}}{\mathrm{TD}}$$

Grain nutrient use efficiency (GUE) was used to assess the accumulation efficiency of Se in the edible parts of crops, specifically in the grain. Where Ct_gCK_ indicates grain Se content in the CK treatment.

$$\mathrm{GUE}=\frac{{\mathrm{Ct}}_{g}-{\mathrm{Ct}}_{\mathrm{gCK}}}{\mathrm{TD}}$$

The recovery rate of Se in the production of polished rice and refined flour was calculated as follows:

$$\mathrm{Recovery rate}=\frac{C_{i}}{\mathrm{Total}\;\mathrm{Se}}$$

Where C_i_ represents Se concentration in the inner fraction of cereals (e.g. polished rice and refined flour).

The estimate of daily Se intake (EDI, μg person^−1^ day^−1^) was calculated according to the following equation:

EDI=C∗M

Where C and M represent Se concentration and cereal consumption, respectively.

### 2.10 Statistical analysis

All data are expressed as mean ± standard deviation (SD) and statistical analyses were performed using SPSS Statistics software (version 26.0, IBM). The normality of variable distribution and the homogeneity of variances were assessed using the Shapiro–Wilk and Levene tests, respectively (α = 0.05). Statistically significant differences (*p* < 0.05) between treatments means were determined by one-way analysis of variance (ANOVA), followed by a post hoc Tukey test.

## 3 Results and discussion

### 3.1 Characterization of Se NPs

**Fig. 1:**
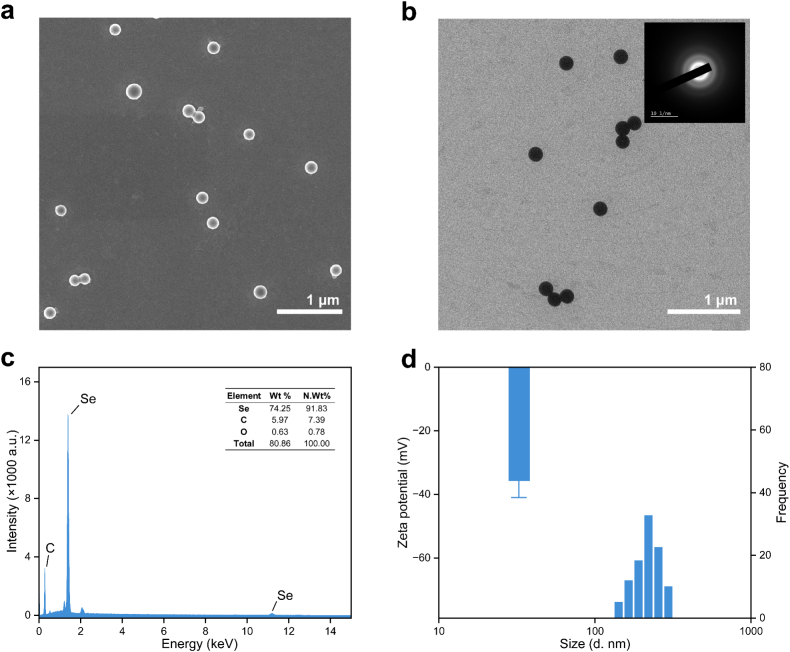
Characterization of the synthesized Se NPs. The representative SEM (a) and TEM image (b) (Inset of TEM image: SAED pattern), and there corresponding energy dispersive X-ray (EDS) spectrum (c). Zeta potential and hydrodynamic diameter of the Se NPs (d).

The morphology of the synthesized Se NPs was characterized using SEM and TEM. As shown in Fig. 1a and b, the NPs exhibited a spherical morphology with smooth surface, with an average particle size of approximately 200 nm. TEM images further confirmed good dispersion of the particles. Elemental analysis via EDS verified the composition as Se NPs with minimal impurities (Fig. 1c). DLS measurements revealed an average hydrodynamic diameter of 219 nm (Fig. 1d), with a negative Zeta potential of 35.6 mV indicating good colloidal stability. Additionally, the SAED pattern (inset of Fig. 1b) exhibited a diffused ring configuration, supported the amorphous structure of the Se NPs.

### 3.2 Agronomic traits of crops

**Fig. 2:**
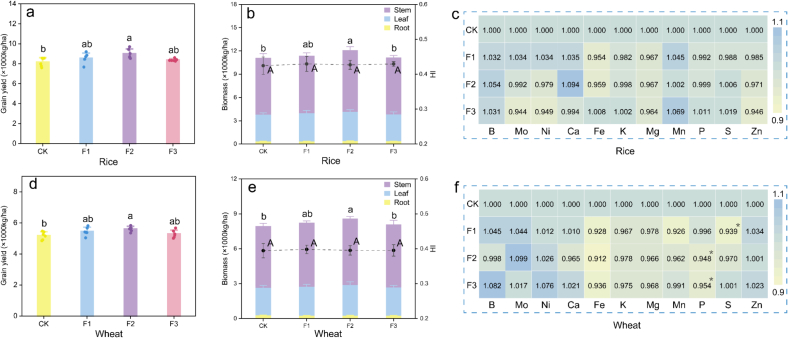
Grain yield (a, d), biomass of stem, leaf and root (b, e). Elemental homeostasis of cereals (c, f). A different letter indicates a significant difference at *p* < 0.05 between groups. * indicates a significant difference at *p* < 0.05 compared with the control. F1, F2, and F3 represent the late heading stage, booting stage, and filling stage, respectively.

Evaluating crop biomass and yield is critical for measuring crop growth and the impact of fertilization on overall agricultural productivity. In this study, foliar application of Se NPs influenced crop yields differently across treatments. As shown in Fig. 2a and d, in the CK treatment, the average grain yields of rice and wheat were 8167 ± 436 kg/ha and 5172 ± 256 kg/ha, respectively. Following Se NPs application, rice yields increased to a range of 8372 ± 123 to 9002 ± 473 kg/ha, while wheat yield rose to 5279 ± 251 to 5604 ± 167 kg/ha. Among the treatments, the booting stage application (F2) resulted in the highest yield increase for both rice and wheat, achieving gains of 10.22 % and 8.35 %, respectively. The effects of exogenous Se applications on crop yield have been widely documented. Previous studies reported yield increases in rice with appropriate doses ranging from 8.24 to 14.65 %(Luo et al., 2020) and with some as high as 31.1 %(Chen, Jiang, et al., 2024). A global meta-analysis by Yan et al.(Yan et al., 2024) revealed that different Se application rates positively impacted wheat yield, with increases reaching up to 7.76 %.

Additionally, the straw biomass of rice and wheat was also significantly affected by Se application. As shown in Fig. 2b and e, the F2 treatment led to a significant increase in straw biomass for both rice and wheat, by 8.92 % and 7.93 %, respectively. While other treatments showed no significant differences. Furthermore, the harvest index (HI) (Fig. 2b and e) parameter was used to evaluate the crops' ability to convert nutrients into economic products. Across all treatments, there were no significant changes in the HIs of both rice and wheat, suggesting that the exogenous Se did not alter crop production efficiencies. It is noteworthy that rice exhibited a higher HI (0.428) than wheat (0.396) (*p* < 0.001), indicating that rice is more efficient than wheat in biomass allocation, directing a greater proportion of photosynthetic products towards its economic yield (i.e. grain).

### 3.3 Cereal nutritional quality and elemental homeostasis

To evaluate the nutritional quality of the cereals under different fertilization timing, we measured the contents of soluble sugars, starch, protein, phytic acid, and mineral elements (Table 1, Fig. 2c and f). The results showed that exogenous Se application had no significant effect on the proportions of soluble sugars, starch, protein, or phytic acid in rice and wheat grains, but their absolute amounts per unit area increased because grain yield was higher (Fig. 2). Consequently, the relative proportions remained stable, whereas the total constituent pools expanded. These results suggest that these quality traits are insensitive to application timing but responsive to the total Se content. Similarly, studies have reported that exogenous Se increased cereal quality such as starch and protein (Chen et al., 2024; Ismail, Nawaz, Shehzad, Haq, & Ashraf, 2024), which is consistent with our conclusion. Comparatively, rice grains contained lower levels of soluble sugars (47.30 mg/g), protein (8.24 %), and phytic acid (7.09 mg/g) than wheat, which exhibited 77.34 mg/g soluble sugar, 13.14 % protein, and 13.49 mg/g phytic acid. In contrast, rice grains had significantly higher starch content (817.72–860.44 mg/g) compared to wheat (615.71–646.93 mg/g). Notably the protein content remained consistently higher in wheat (13.01–13.32 %) than in rice (8.18–8.26 %) across treatments.

**Table 1:** Soluble sugars, starch, protein and phytic acid of cereals. A different letter indicates a significant difference at p < 0.05 between groups. F1, F2, and F3 represent the late heading stage, booting stage, and filling stage, respectively.

		Soluble sugars (mg/g)	Starch (mg/g)	Protein (%)	Phytic acid (mg/g)
Rice	CK	47.37 ± 3.06a	840.39 ± 35.2a	8.26 ± 0.27a	7.08 ± 0.82a
	F1	49.19 ± 1.26a	860.44 ± 77.46a	8.26 ± 0.19a	7.34 ± 0.75a
	F2	45.94 ± 2.76a	818.48 ± 62.95a	8.18 ± 0.23a	6.93 ± 0.33a
	F3	46.69 ± 2.74a	817.72 ± 39.68a	8.25 ± 0.21a	7.01 ± 0.25a
Wheat	CK	78.67 ± 5.33a	616.27 ± 23.86a	13.01 ± 0.47a	13.67 ± 1.26a
	F1	77.46 ± 3.52a	646.93 ± 25.48a	13.07 ± 0.2a	13.87 ± 1.22a
	F2	75.05 ± 3.27a	624.45 ± 41.47a	13.14 ± 0.35a	13.25 ± 0.49a
	F3	78.18 ± 3.12a	615.71 ± 25.14a	13.32 ± 0.38a	13.18 ± 0.39a

Additionally, the analysis of mineral nutrients in cereals (Fig. 2c and f) showed that exogenous Se had no significant effects on the mineral nutrient content in rice grains. In wheat grains, no significant changes were observed in most elements, with the exception of P, which showed a reduction under the F2 and F3 treatments and S, which decreased under the F1 treatment. These findings are consistent with previous studies where Se spraying had little relationship with Fe, Cu, Zn, etc.(Di et al., 2023) A possible explanation is that at this level, Se is insufficient to trigger antagonistic interactions between elements. Moreover, due to the chemical similarities between S and Se, plants may incorporate Se into the sulfur assimilation pathway. (Schiavon and Pilon-Smits, 2017a, b) This biochemical overlap allows Se to be assimilated without disrupting the uptake or metabolism of the other mineral nutrients. In conclusion, exogenous Se simultaneously increased the contents of soluble sugars, protein and phytic acid, while preserving the mineral nutrient homeostasis, highlighting the compatibility of Se biofortification with overall cereal nutrient balance.

### 3.4 Se levels in crop tissues

**Fig. 3:**
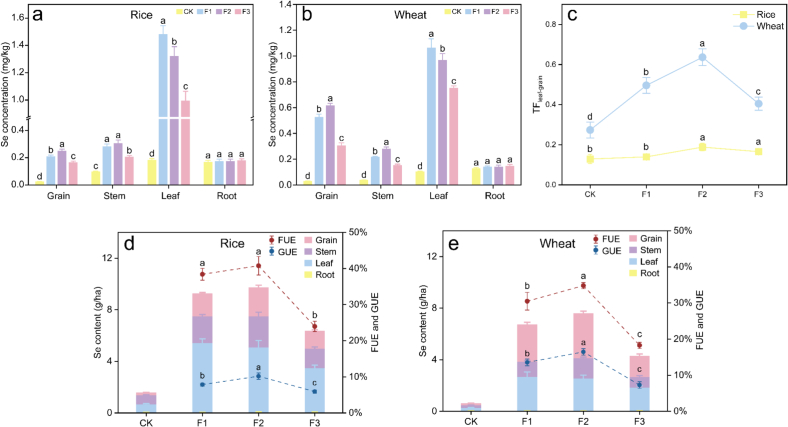
Se levels in crops. Se concentration in different tissues of rice (a) and wheat (b). Translocation factors from leaf to grain (c). Se content in crops per hectare and the response fertilizer use efficiency (FUE) and grain nutrient use efficiency (GUE) (d, e). A different letter indicates a significant difference at p < 0.05 between groups. F1, F2, and F3 represent the late heading stage, booting stage, and filling stage, respectively.

To assess the effectiveness of Se biofortification, Se concentrations were measured across various tissues of rice and wheat. Following foliar Se application, a significant increase in the Se accumulation was observed in both crops (Fig. 3a and b). Specifically, in the CK, the Se concentrations in rice grain, stem, and leaf were 23.14, 95.74, and 181.03 μg/kg, respectively. These increased to 164.10–247.26, 204.72–303.84, and 993.70–1318.45 μg/kg under se treatments, representing 6.1–9.7, 1.1–2.2, and 4.5–6.3 fold increases respectively. Notably, the Se concentrations in rice roots (166.41–178.25 μg/kg) showed no significant change across treatments. Similarly, wheat grain, stem, and leaf Se concentration increased from the CK values of 27.80, 37.36, and 101.84 μg/kg, respectively, to 303.29–612.84, 150.23–278.50, and 750.15–1060.11 μg/kg, corresponding to increases of 9.9–21.0, 3.0–6.5 and 6.4–9.4 times, respectively. Roots Se concentration in wheat (126.68–144.76 μg/kg) also remained unchanged. Among different application timings, Se application at the booting stage (F2) led to the highest grain Se concentration in the both crop, aligning with the findings of Huang et al.(Huang, Ding, Guo, Zhang, & Wang, 2018). Notably, wheat exhibited a higher grain Se concentration than rice, likely attributable to its greater protein content (Table 1), given that Se in grains is primarily present in the speciation of SeMet, which is incorporated into proteins (Schiavon and Pilon-Smits, 2017a; Y. Wang et al., 2023; X. Wang, Hussain, et al., 2025; Terry, Zayed, De Souza, & Tarun, 2000) (detailed below). TF values (Fig. 3c) further demonstrates this disparity; wheat showed significantly higher TFs_leaf - grain_ (0.27–0.64) compared to rice (0.13–0.19) (*p* < 0.01), indicating a more efficient Se allocation from leaves to grains in wheat. Additionally, different fertilization timings affect the distribution of Se within plant. In rice, the TF_leaf - grain_ increased from 0.13 in the CK to 0.19 (F2) and 0.17 (F3), While in wheat it rose from 0.27 (CK) to 0.50 (F1), 0.64 (F2), and decreased to 0.40 (F3). Both rice and wheat had the highest TF_leaf - grain_ under F2, which is one of the reasons for the highest grain Se concentration observed in F2. Differences in TFs across fertilization stages may stem from timing-dependent Se NP dissolution, metabolic conversion, and source–sink transport dynamics. With early (F1) application, Se NPs are progressively metabolized to selenoamino acids and non-specifically incorporated into leaf structural proteins(Y. Wang et al., 2023), reducing their remobilization potential. With filling stage (F3) application, the time for biotransformation may be insufficient, limiting uptake and phloem loading. As a result, TF tends to be lower at F1 and F3, while mid-stage (F2) application aligns dissolution/assimilation with active grain filling and strong sink demand(Giordano, Sadras, & Lollato, 2023), yielding a higher TF.

Taking into account the total biomass of the crops, we calculated the Se content per hectare (Fig. 3d and e). Under Se treatments, the Se content in rice was 6.36–9.72  g/ha, with fertilizer use efficiency (FUE) of 38.4 % (F1), 40.8 % (F2) and 24.0 % (F3). Most Se was stored in the leaves (51.7–58.0 %). In contrast, for wheat Se content ranged from 4.27 to 7.58  g/ha, with FUE of 30.5 % (F1), 34.8 % (F2) and 18.3 % (F3), and Se was primarily stored in the grains (37.6–45.3 %) and leaves (33.4–42.6 %), this is consistent with the findings of Di et al. (2023). The FUE of rice was significantly higher than that of wheat (*p* < 0.01), indicating that rice can absorb Se more efficiently under the same fertilizer application rate. Furthermore, considering that only the Se in the edible part, the grains, can be ingested by humans, we evaluated the grain nutrient use efficiency (GUE). The results showed that wheat exhibited significantly higher GUE (7.3–16.5 %) compared rice (5.9–10.2 %) (*p* < 0.01), indicating a superior ability to translocate and accumulate Se in edible grain fractions. Conversely, rice retained a larger proportion of absorbed Se in non - edible parts such as roots, stems and leaves.

### 3.5 Biodistribution of Se NPs in crops

**Fig. 4:**
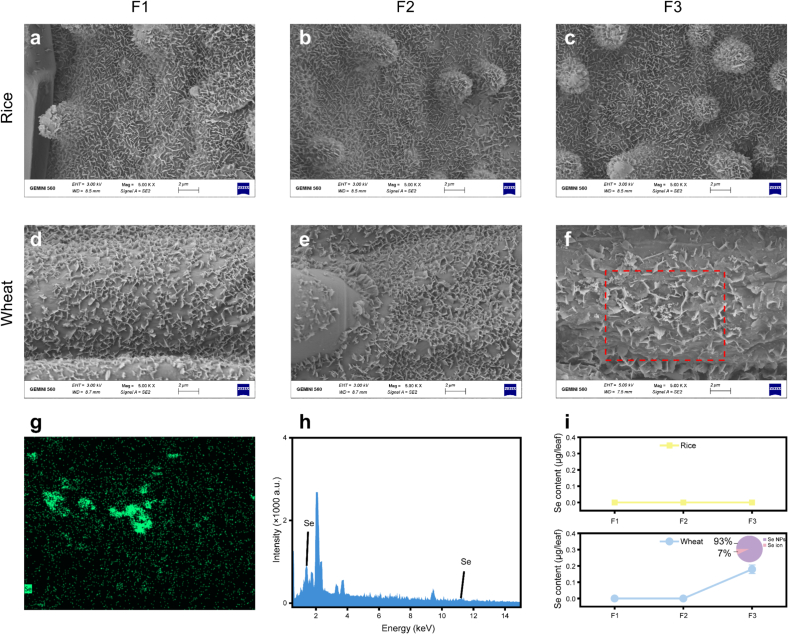
Representative SEM images of rice (a-c) and wheat (d-f) leaf surface at maturity. EDS analysis (g, h) of the red-boxed area in Figure f. And Se concentration in leaf eluate (i), pie chart representing the Se form composition (ionic and particulate form) in eluate under F3 treatment. F1, F2, and F3 represent the late heading stage, booting stage, and filling stage, respectively. (For interpretation of the references to colour in this figure legend, the reader is referred to the web version of this article.)

To gain deeper insights into the transformation and bioaccumulation of Se NPs, their accumulation in leaves and grains of mature plants was assessed. SEM images showed that the particles remained on the surface of the wheat leaves under F3 treatment (Fig. 4f), with EDS analysis confirming these surface particles as Se NPs (Fig. 4g,h). No visible nanoparticle residues were observed under F1, F2, nor in any rice leaf samples across all the treatments. SP-ICP-MS analysis of the leaf eluent indicated that 0.18 μg/leaf of Se remained on the wheat leaf under F3 treatment, with 93 % of this Se present in nanoparticles form (Fig. 4i). In contrast no residual ionic or nano Se was detected under other treatments. This might be the main reason for the low FUE in wheat under F3 treatment, as the delayed application likely hindered the penetration of Se NPs into the leaves, resulting in reduced efficacy and fertilizer loss.

**Fig. 5:**
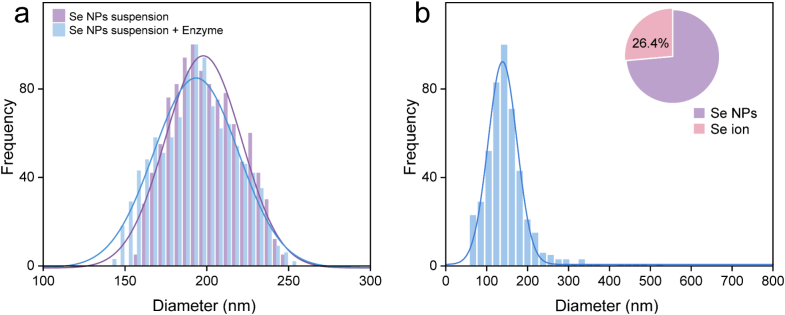
Particle size distribution and frequency of Se NPs suspension and Se NPs treated with Macerozyme R-10 (a). Se form composition (ionic and particulate form) in wheat leaf under filling stage treatment (b).

Quantification of Se nanoparticles in both cereals and leaves revealed consistent trends. As shown in Fig. 5a, the preliminary experiment indicates the satisfactory performance of the detection method. In wheat, particulate Se was detected only in the leaves under the F3 treatment (Fig. 5b), while only ionic Se was detected in the grains and in leaves under treatments. In rice, no Se NPs was detected in any of the treatments. Specifically, under the F3 treatment, 26.4 % of the Se in wheat leaves remained in nano form, with a particle concentration of 4.6 × 10^11^ /kg. These Se NPs ranged in size from 66 to 300 nm, with an average size reduction from the original 200 nm to 142 nm. These findings suggest that even though nanoparticles persist in wheat leaves, they are not transported to the wheat grains. In rice, the complete transformation of the absorbed Se NPs into the ionic Se similarly prevented the nanoparticles accumulation in grains. It is known that Se NPs in plants can be transformed into Se compounds(X. Zhou et al., 2024), and the binding to proteins has been suggested as a mechanism limiting their transport(Schild et al., 2014; Y. Wang et al., 2023). Importantly, the absence of Se NPs in the edible parts provides reassurance regarding food safety in the context of nano-enabled agriculture.

### 3.6 Nutritional profile of Se in cereals

**Fig. 6:**
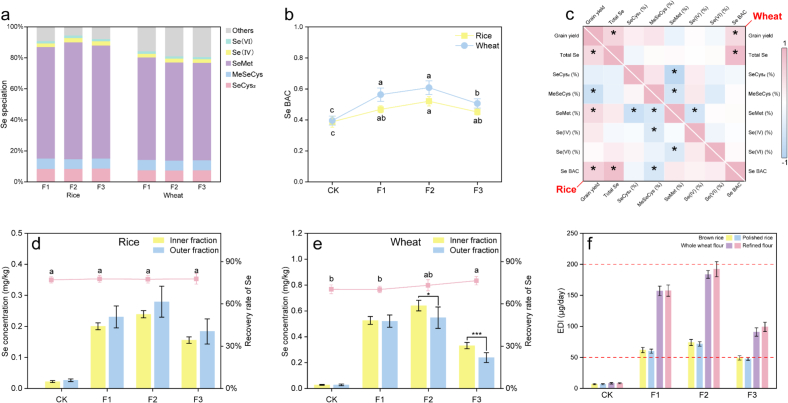
Nutritional profile of se in cereals. Se speciation (a), Se bioaccessibility (b). Correlation analysis of grain yield, total Se concentration, proportion of each Se speciation, and Se BAC (c), % indicates that the value was calculated in percent, *: *p* < 0.05. Se concentration in inner and outer fraction of cereals (d, e). Estimated daily intake of Se in brown rice, polished rice, whole wheat flour and refined flour (f). A different letter indicates a significant difference at *p* < 0.05 between groups. F1, F2, and F3 represent the late heading stage, booting stage, and filling stage, respectively. (For interpretation of the references to colour in this figure legend, the reader is referred to the web version of this article.)

The ultimate objective of present study is the effective fortification of cereals with Se. As discussed earlier, foliar application of Se NPs significantly increase the Se concentration in both rice and wheat grains. However, the nutritional value of Se is determined not only by its total concentration but also by its chemical speciation(Thiry, Ruttens, De Temmerman, Schneider, & Pussemier, 2012). While, SP-ICP-MS confirmed the absence of the nano form of Se in the cereal grains, HPLC-ICP-MS was employed to further characterize the non-nano Se species present in the fortified grains. As illustrated in Fig. 6a, the predominant Se species in both wheat and rice grain was organic Se. Specifically, SeMet accounts for 71.8–75.4 % of the total Se in rice and 62.9–66.1 % in wheat, while inorganic Se (Se(IV) and Se(VI)) constitute less than 4.5 %. Rice exhibits a higher proportion of SeMet than wheat, which may be related to their differing abilities to transport various speciation of Se. Rice more efficiently translocates SeMet into the grains(Carey et al., 2012), whereas wheat excels in Se(VI) transport(Kikkert & Berkelaar, 2013). Although some previous studies suggested that SeCys predominant in rice(Dai et al., 2019; Yeasmin et al., 2025), more evidence supports that SeMet is the predominant form in whether rice or wheat(Di et al., 2023; Y. Wang et al., 2023; Xie, Sun, Li, Shen, & Fang, 2021; Zeng et al., 2023). Under different treatments, the proportions of Se species in both rice and wheat indicating that the exogenous Se did not disrupt the Se metabolism. This stability is consistent with the understanding that, under non-Se-stress conditions, the Se metabolism in non-accumulators plants proceeds nonspecifically via sulfur metabolic pathways(Chauhan et al., 2019; Trippe & Pilon-Smits, 2021), without including new or specialized metabolic pathways.

Se BAC in grains was also evaluated. As shown in Fig. 6b, under various treatments, the Se BAC in rice was 38.8–52.1 %, which was lower than that in wheat at 39.6–60.8 % (*p* < 0.01). Compared with the CK, although the Se speciation in rice and wheat did not change significantly under Se NP treatments, an increase in Se BAC was observed, suggesting improved Se nutritional quality. Correlation analysis (Fig. 6c) revealed a positive relationship between total Se concentration and Se BAC in both rice and wheat. Interestingly, while Se BAC correlated positively with concentrations of organic Se (Fig. S2), it did not correlate with the proportion of organic Se, except for MeSeCys in rice (Fig. 6c). Previous studies attributed the positive correlation between Se BAC and organic Se to the effects of organic Se, potentially overlooking the more significant influence of total Se concentration. However, the bioaccessibility assessed by PBET has limitations, and the bioavailability and functions of various seleno-metabolites still requires validation through in vivo studies in animal models.

The distribution of Se in cereals grains is crucial for its retention during food processing. In our study, we analyzed the distribution of Se in rice and wheat grains, and recovery rate is used to assess nutrient retention after processing (Lian et al., 2023). As shown in Fig. 6d, rice displayed no significant difference in Se concentration between the inner and outer fractions. For example, Se levels ranged from 22.28 vs. 26.54 μg/kg in CK, 200.24 vs. 230.50 μg/kg in F1, 239.18 vs. 279.60 μg/kg in F2, and 155.84 vs. 183.81 μg/kg in F3 (inner vs. outer fractions, respectively). The overall Se recovery rate remained stable at 77.1–77.8 %. By contrast, wheat showed a variable distribution pattern (Fig. 6e). At CK, Se levels were similar between fractions (27.84 vs. 27.73 μg/kg). With foliar Se application, inner-fraction concentrations became higher: 525.71 vs. 520.52 μg/kg in F1, 640.78 vs. 547.65 μg/kg in F2, and 331.03 vs. 238.55 μg/kg in F3 (inner vs. outer). These differences correspond to 1.17-fold and 1.39-fold enrichment of the inner fraction under F2 and F3, respectively. Consistently, the Se recovery rate increased from 70.3 % (CK) and 70.2 % (F1) to 73.2 % (F2) and 76.5 % (F3), suggesting that delayed foliar application enhances Se retention in the edible portion of wheat grain. A possible explanation is that Se NPs are progressively metabolized into selenoamino acids, which are non-specifically incorporated into proteins(Schiavon and Pilon-Smits, 2017b; Y. Wang et al., 2023; X. Wang, Hussain, et al., 2025). Se applied early is assimilated into leaf proteins and retained until grain filling, when it is remobilized together to the grain. Whereas Se applied during grain filling is converted to amino acids and can be directly translocated to the grain, the intense mid- to late-filling deposition of storage proteins in the endosperm drives marked Se accumulation in the inner fraction(Zhang et al., 2021, Zhang et al., 2024). By contrast, in rice this effect is muted because grain filling is shorter (∼10–15 d vs. ∼30–40 d in wheat) and grain protein content is lower (Table 1).

According to WHO recommendations, the daily Se intake for adults should be between 50 and 200 μg. Under CK treatment, the EDI from brown rice, polished rice, whole wheat flour, and refined flour was below 8.4 μg/day, significantly lower than the recommended intake. Following Se fortification, treatments except for rice under F3 treatment provided sufficient Se to improving Se nutritional status through cereal consumption.

## 4 Conclusion

This study suggests that foliar application of Se NPs is an effective strategy for Se biofortification of rice and wheat. The delivery potential of targeted Se in cereals is closely related to the fertilization time. Application of Se NPs at the booting stage in rice and wheat was optimal for maximizing both fertilizer use efficiency and grain nutrient use efficiency, while application during the filling stage was most effective in enhancing Se recovery in refined flour. In terms of health implications, our results showed that Se NPs are not expected to bioaccumulate in edible parts, thus avoiding public concerns about food safety from nano-enabled agriculture.

Nevertheless, this study was conducted in a single season at one location with one Se NPs formulation and dose. Multi-season, multi-location trials spanning diverse soils, climates and genotypes are needed to validate the timing recommendations and establish dose–response ranges.

## CRediT authorship contribution statement

**Xin Wang:** Writing – original draft, Methodology, Investigation. **Bilal Hussain:** Formal analysis, Data curation. **Jiapan Lian:** Methodology, Investigation. **Xiaoping Xin:** Writing – review & editing, Investigation. **Tong Zou:** Investigation. **Xiwei Huang:** Investigation. **Liping Cheng:** Investigation. **Hongyu Yu:** Investigation. **Zhenli He:** Supervision, Methodology, Investigation. **Xiaoe Yang:** Writing – review & editing, Supervision, Methodology, Funding acquisition.

## Declaration of competing interest

The authors declare that they have no known competing financial interests or personal relationships that could have appeared to influence the work reported in this paper.

## Data Availability

Data will be made available on request.

## Appendix A Supplementary data

Supplementary material Image 1
