# Supplementary material for: Time-dependent application strategies of selenium Nanofertilizer to enhance biofortification in rice and wheat
Source: Food Chem X. 2025 Oct 16;31:103174. doi: 10.1016/j.fochx.2025.103174 (PMC12590002; doi:10.1016/j.fochx.2025.103174)
Supplement: Supplementary file 1 — Supplementary material [file mmc1.docx]

*Supplementary Information for*

**Time-Dependent Application Strategies of Selenium Nanofertilizer to Enhance Biofortification in Rice and Wheat**

Xin Wang ^a^, Bilal Hussain ^a^, Jiapan Lian ^a,b,*^, Xiaoping Xin, ^c^ Tong Zou ^a^, Xiwei Huang ^a^, Liping Cheng ^a^, Hongyu Yu ^a^, Zhenli He ^d^, Xiaoe Yang ^a,^^*^

^a^ Ministry of Education (MOE) Key Laboratory of Environmental Remediation and Ecosystem Health, College of Environmental and Resources Sciences, Zhejiang University, Hangzhou, 310058, China

^b^ State Key Lab for Conservation and Utilization of Subtropical Agro-Bioresources, Guangxi Key Lab of Sugarcane Biology, Guangxi University, Nanning 530004, China

^c^ School of Natural Resources, Division of Plant Sciences and Technology, University of Missouri, MO, 65211 USA

^d^ Department of Soil, Water and Ecosystem Sciences, Indian River Research and Education Center, University of Florida-IFAS, Fort Pierce, FL 34945, USA

**^*^Corresponding Author:** Xiaoe Yang; Jiapan Lian

**E-mail:** [xeyang@zju.edu.cn](mailto:xeyang@zju.edu.cn) (X.E. Yang); Ljiapan@zju.edu.cn (J.P. Lian)

**Full Postal Address:** No. 866 Yuhangtang Road, College of Environmental and Resources Sciences, Zhejiang University, Hangzhou, 310058, China

**Phone/Fax:** +86 0571 88982907.

**Contents:**

**Table S1.** Soil physical and chemical properties.

**Table S2.** Operating parameters of SP-ICP-MS.

**Figure S1.** Meteorological conditions at the field site during the experimental period.

**Figure S2.** Correlation analysis of grain yield, total Se concentration, concentration of each Se speciation, and Se BAC.

**Number of pages: 4**

**Number of figures: 1**

**Number of tables: 1**

**Table S1.** Basic characteristics of the experimental soil (n=6).

| **Characteristics** | **Rice** | **Wheat** |
| --- | --- | --- |
| Texture | Loam | Loam |
| pH | 5.4 ± 0.1 | 5.4 ± 0.1 |
| CEC (cmol^+^·kg^-1^) | 12.6 ± 1.1 | 12.4 ± 0.8 |
| Total Se (mg·kg^-1^) | 0.16 ± 0.04 | 0.16 ± 0.03 |
| Organic matter (g·kg^-1^) | 25.24 ± 2.78 | 24.16 ± 3.24 |
| Total N (g·kg^-1^) | 1.20 ± 0.11 | 1.23 ± 0.18 |
| Total P (g·kg^-1)^ | 1.14 ± 0.13 | 1.16 ± 0.20 |
| Total K (g·kg^-1)^ | 18.24 ± 2.08 | 19.10 ± 1.86 |

**Table S2.** Operating parameters of SP-ICP-MS.

| Parameter | Value |
| --- | --- |
| Detection system | EXPEC 7350 |
| Introduction system | 200μL Nebulizer - Scott Spray Chamber |
| Sample flow rate | 50 μL/min |
| Analyte monitored | 80 nm ^197^Au NPs |
| Analysis time | 120 - 600s |
| Transport efficiency for quantity | 13.54% |
| Transport efficiency for particle size | 9.36% |
| Analyte | ^78^Se |
| LOD in size (Au) | 10 nm |
| LOD in mass (Au) | 0.1 ug/L |
| LOD in particle number | 10³ /mL |


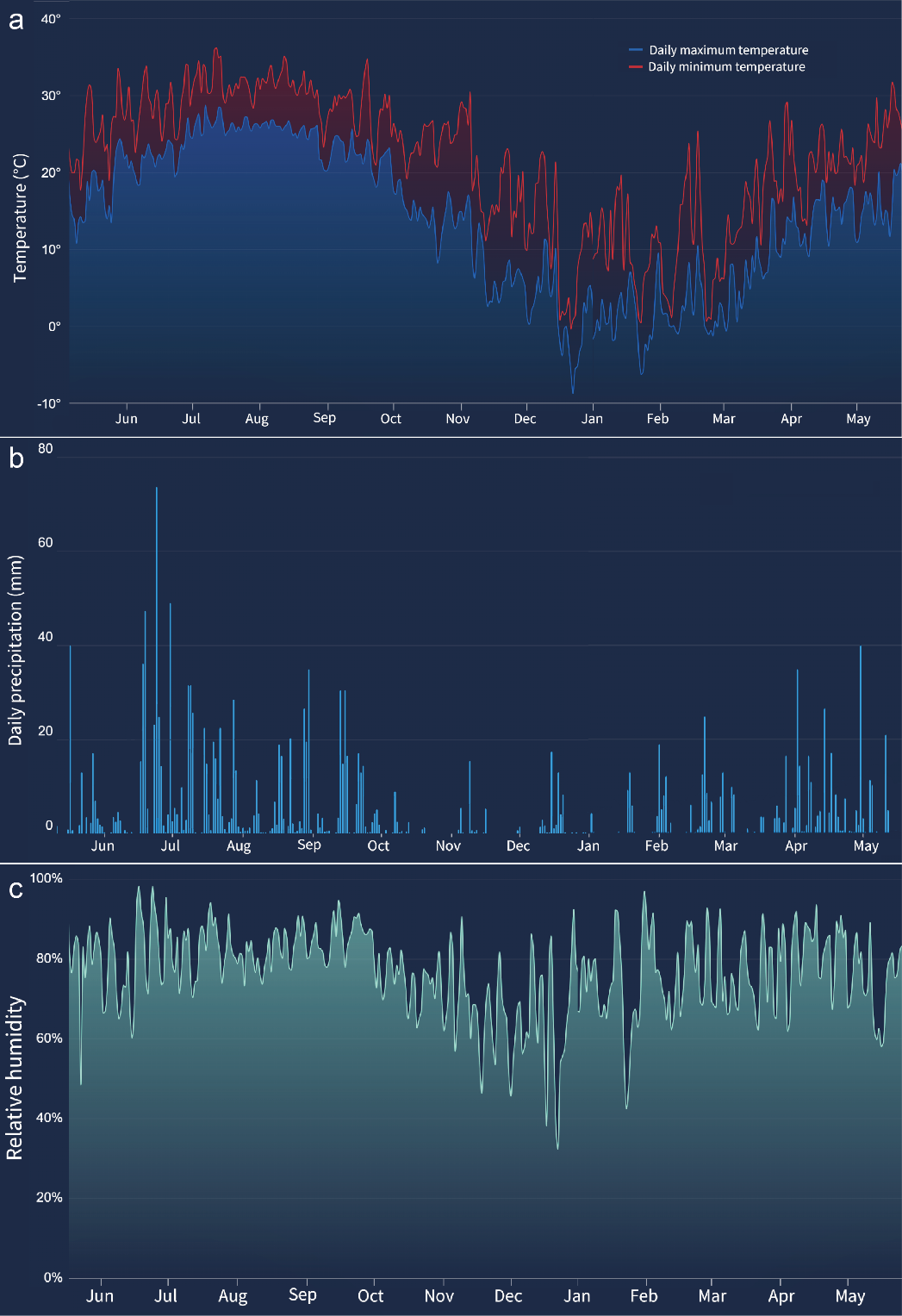


**Figure S1.** Meteorological conditions at the field site during the experimental period (June–May). (a) Daily maximum and minimum air temperature; (b) daily precipitation; (c) daily mean relative humidity.


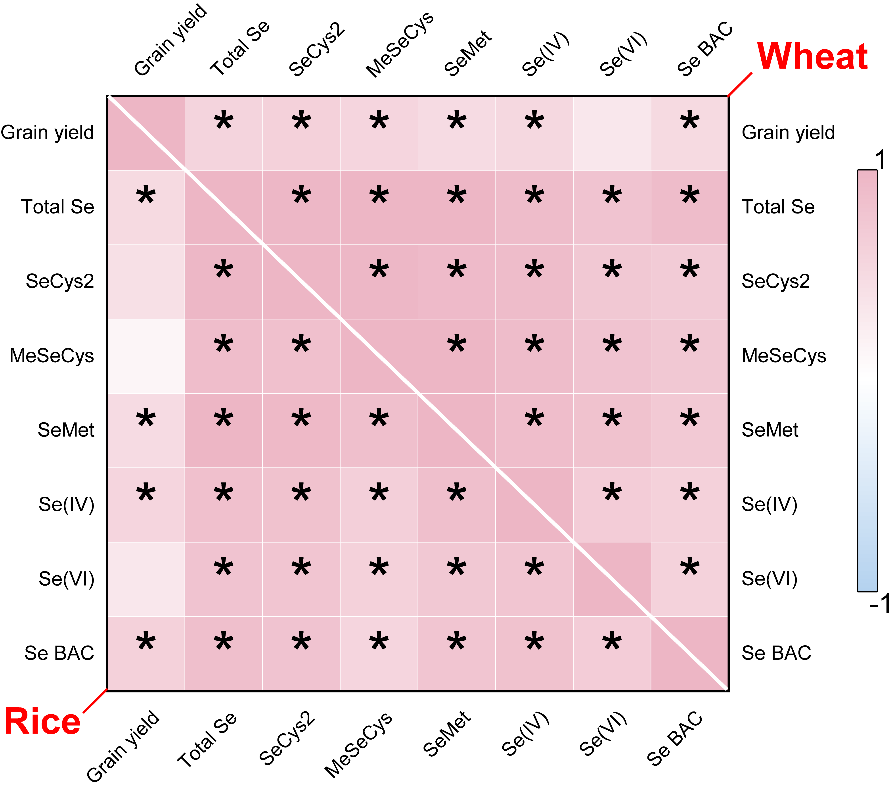


**Figure S2.** Correlation analysis of grain yield, total Se concentration, concentration of each Se speciation, and Se BAC. *: *p* < 0.05.
